# *RYR1* causing distal myopathy

**DOI:** 10.1002/mgg3.338

**Published:** 2017-10-04

**Authors:** Ruple S. Laughlin, Zhiyv Niu, Eric Wieben, Margherita Milone

**Affiliations:** ^1^ Department of Neurology Mayo Clinic Rochester Minnesota; ^2^ Department of Laboratory Medicine and Pathology Mayo Clinic Rochester Minnesota; ^3^ Department of Clinical Genomics Mayo Clinic Rochester Minnesota; ^4^ Department of Biochemistry and Molecular Biology Mayo Clinic Rochester Minnesota

**Keywords:** Centronuclear, distal myopathy, myopathy, *RYR1*

## Abstract

**Background:**

Congenital myopathies due to ryanodine receptor (*RYR1*) mutations are increasingly identified and correlate with a wide range of phenotypes, most commonly that of malignant hyperthermia susceptibility and central cores on muscle biopsy with rare reports of distal muscle weakness, but in the setting of early onset global weakness.

**Methods:**

We report a case of a patient presenting with childhood onset hand stiffness and adult onset progressive hand weakness and jaw contractures discovered to have two variants in the *RYR1* gene.

**Results:**

The patient manifested with distal upper limb weakness which progressed to involve the distal lower limb, proximal upper limb, as well as the face in addition to limited jaw opening. Creatine kinase was mildly elevated with EMG findings supporting a myopathy. Muscle biopsy showed features consistent with centronuclear myopathy. Whole exome sequencing revealed a novel heterozygous pathogenic variant in *RYR1* (c.12315_12328delAGAAATCCAGTTCC, p.Glu4106Alafs*8), and a heterozygous missense variant (c.10648C>T, p.Arg3550Trp) of unknown significance in compound heterozygous state.

**Conclusion:**

We expand the spectrum of *RYR1*‐related myopathy with the description of a novel phenotype in an adult patient presenting with hand weakness and suggest considering *RYR1* analysis in the diagnosis of distal myopathies.

## Introduction

Pathogenic mutations in the ryanodine receptor 1 (*RYR1*) gene located on chromosome 19q13.2 have a spectrum of clinical manifestations that can present from intrauterine life to adulthood. Early descriptions of *RYR1*‐related myopathy were associated with autosomal dominant inheritance producing a clinical phenotype of malignant hyperthermia and central core disease (Quane et al. 1993; McCarthy et al. 2000; Zhou et al. 2006). Since that time, the disease spectrum due to *RYR1* mutations has evolved and is now recognized as one of the most common congenital myopathies (Jungbluth et al. 2002). The morphological changes on muscle biopsy span from central cores to multi‐minicores, central nuclei, congenital fiber type disproportion, or may be absent (Jungbluth et al. 2016). In addition, *RYR1*‐related myopathies can be inherited also in an autosomal recessive manner, although more frequently with a dominant trait (Jungbluth et al. 2002; Clarke et al. 2010; Bevilacqua et al. 2011; Snoeck et al. 2015).

Here we describe a patient with distal myopathy found to be a compound heterozygous for a novel pathogenic variant in *RYR1* and an additional missense variant of unknown significance in the same gene, expanding the spectrum of *RYR1*‐related myopathy.

## Case Report

A 54‐year‐old RH male of full Polish descent was referred to our neuromuscular clinic citing a 1.5 year history of progressive upper limb weakness. He first noted weakness in his hands, followed by lower face weakness. The weakness progressed proximally in upper limbs, with reduced ability to perform any heavy lifting. He also noted that speaking for a long time had become more difficult due to the front of his mouth tensing up with prolonged talking and limited jaw excursion. Additionally, he stated that routine dental appointments were markedly difficult as he could not open his mouth widely, nor for a long time, such as was required during dental visits. He did not notice any lower limb symptoms and was still able to climb two flights of stairs without difficulty. He had no myalgia or exercise‐induced cramps.

Although not a primary complaint, on further questioning, he recalled that as a child he was less “strong” than his peers. In childhood he developed cold‐induced stiffness of his hands, which continues to date. He denied sensory complaints, fasciculations, visual symptoms, dysphagia, or fluctuations in his symptoms. He had no systemic complaints or rash. Past medical history was notable for only one prior surgery >20 years ago requiring general anesthesia without complications, but could not recall if volatile anesthetics were used. He denied any episode of rhabdomyolysis or urine discoloration.

**Figure 1:**
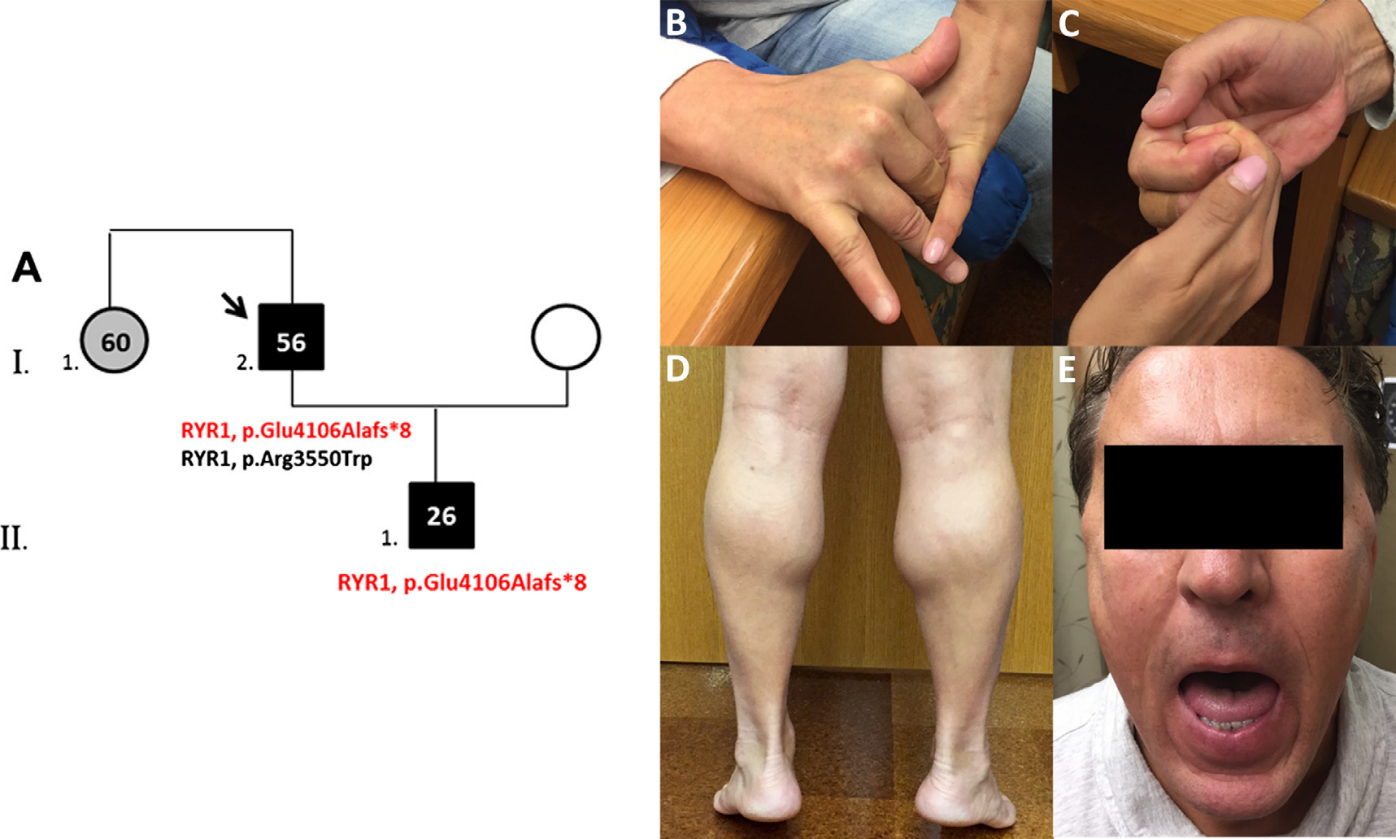
Family pedigree and patient. (A) The arrow indicates the proband. Gray color denotes the individual with reported muscle weakness but not available for examination. Patient's photographs indicate (B) severe weakness of the finger extensors (C) with spared flexors, (D) calf muscle enlargement, and (E) jaw contracture limiting opening of the mouth.

Family history was notable for a son with diffuse myalgias, elevated creatine kinase (CK) level without overt weakness and an older sister who developed diffuse but proximal predominant weakness around age 16 (Fig. 1A). Her weakness resulted in progressive inability to climb stairs or lift things overhead. By age 60, she was nonambulatory. Parents were reportedly asymptomatic. There was no family history of sudden death, reported anesthetic complications in family members, and no consanguinity.

Neurological examination revealed jaw contracture. Mild facial weakness was demonstrated without ophthalmoplegia and normal neck extensor and flexor strength. Moderate distal and mild proximal upper limb weakness was also identified. No muscle atrophy was noted; rather, prominent calf bulk was identified, also present in his affected sister as per history (Fig. 1B–E). Reflexes were decreased in the upper limbs. Lower limb strength and reflexes were normal. Tone and sensory examination and gait were normal. No clinical myotonia could be elicited. Despite full lower limb strength, the patient had mild impairment on heel and toe walking possibly suggesting subtle distal lower limb weakness. No scoliosis was noted.

Local laboratory evaluation showed a mildly elevated creatine kinase to 446 U/L (normal <336). MRI of the cervical spine performed locally to exclude a structural cause for arm weakness was essentially normal. Electromyography showed an electrophysiological severe myopathy with mild to moderate fibrillation potentials and short duration motor unit potentials diffusely. Cardiorespiratory examination was normal. When seen in follow‐up 1 year later, the patient complained of progressive finger weakness and burning in his feet with follow‐up testing confirming a limited distal small fiber neuropathy. Examination of the upper limb revealed little changed, but lower limb examination showed new mild lower limb weakness distally when compared to the initial visit.

**Figure 2:**
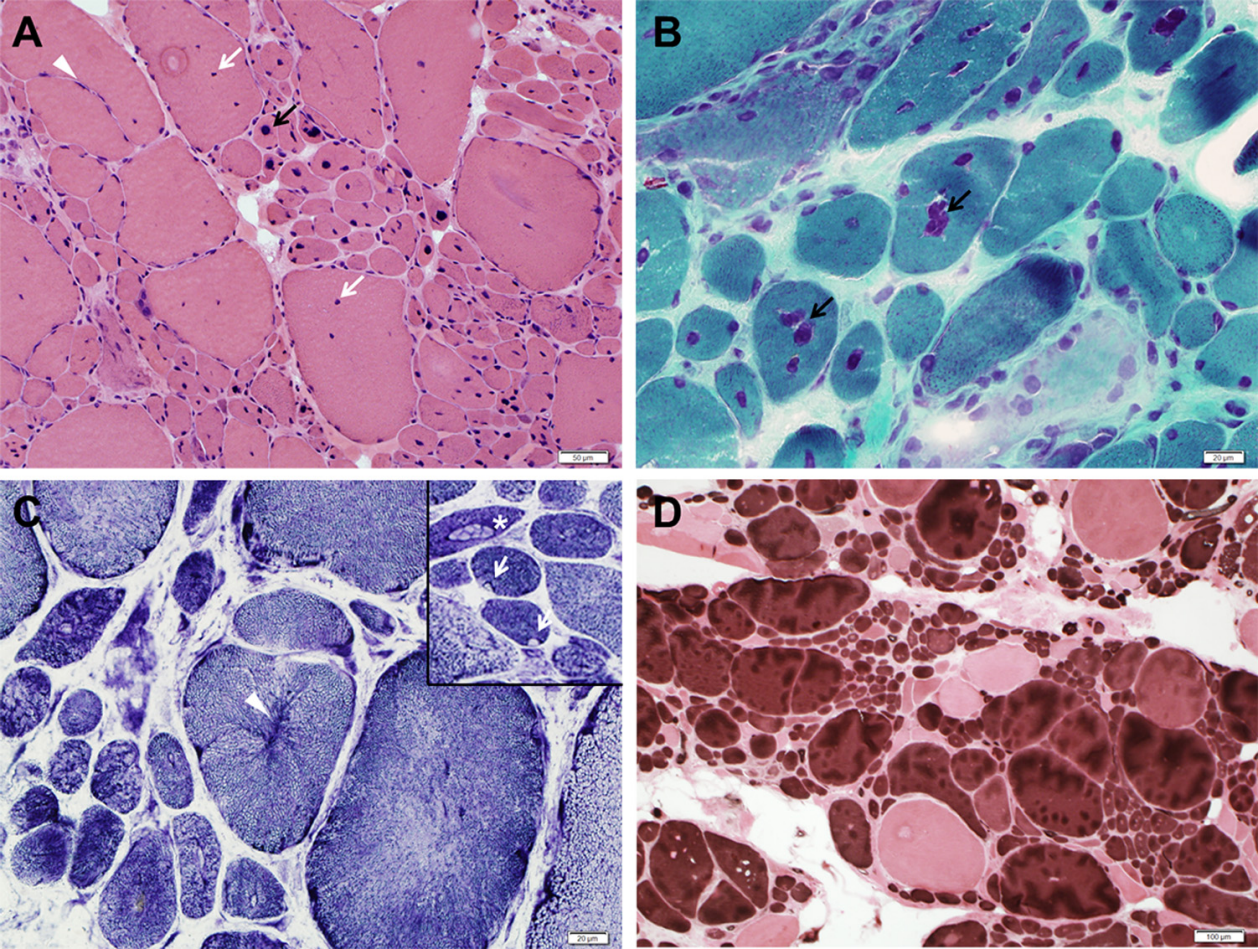
Muscle biopsy. (A) There is muscle fiber size variability with numerous fibers being atrophic and a few hypertrophic, muscle fibers subdividing by splitting (arrow head), fibers with internalized nuclei (white arrow), and numerous fibers with single central nucleus (black arrow) (hematoxylin–eosin). (B) In several fibers the internalized nuclei are closely clustered (arrows) (modified Gomori trichrome). (C) Occasional fibers show radial distribution of sarcoplasmic strands (arrow head) and rare fibers show subsarcolemmal focal decreases of enzyme reactivity (arrows) with small core‐like structures in some fibers (asterisk), as seen in right upper corner (NADH). (D) There is type 1 fiber (dark fibers) predominance and most atrophic fibers are type 1 (ATPase pH 4.3).

Muscle biopsy of the biceps brachii showed several structural abnormalities (Fig. 2). There was muscle fiber size variability and fibers subdividing by splitting. Almost all fibers had multiple internalized nuclei, scattered or clustered, or a single central nucleus. In NADH dehydrogenase‐reacted sections, only rare fibers showed radial distribution of sarcoplasmic strands, rare other fibers harbored focal loss of enzyme reactivity, occasionally featuring tiny single core‐like structures. In ATPase‐reacted sections, the random distribution of histochemical fiber types was altered by fiber splitting, but type 1 fibers were more abundant than type 2 fibers, and the atrophic fibers were mainly type 1. There were no necrotic or regenerating fibers and no inflammatory changes. Caffeine halothane contracture test (CHCT) was not performed.

### Molecular genetic studies

Targeted genetic analysis of genes causative of several distal myopathies (*ANO5*,* BAG3*,* CAV3*,* CRYAB*,* DES*,* DNAJB6*,* DYSF*,* FHL1*,* FLNC*,* GNE*,* LDB3*,* MATR3*,* MYH7*,* MYOT*,* TIA1*,* TTN* exons 307‐312, and *VCP*) by next‐generation sequencing (Prevention Genetics, Marshfield, WI) revealed no pathogenic variants prior to evaluation at our center. Whole exome sequencing (Mayo Clinic, Rochester MN) showed a novel heterozygous pathogenic variant in *RYR1*, c.12315_12328delAGAAATCCAGTTCC, p.Glu4106Alafs*8, and a heterozygous missense variant of unknown significance (VUS) in the same gene (c.10648C>T, p.Arg3550Trp). A hemizygous VUS in *DMD* (p.Leu2078Phe) was also detected. The proband's symptomatic son carries only p.Glu4106Alafs*8 (Fig. 1A). DNA from the proband's affected sister and asymptomatic son was not available for analysis. The proband's *RYR1* variants, as well as the *DMD* variant, were confirmed by clinical testing in a commercial laboratory (EGL Genetics, Tucker, Georgia; reported in ClinVar). Diagnostic testing in the same commercial laboratory excluded the presence of deletions and duplications in genes causative of distal myopathies and in *RYR1* by comparative genomic hybridization (CGH).

## Discussion

The *RYR1* encodes for the calcium release channel within the sarcoplasmic reticulum of muscle. This function enables the calcium release required for tropomyosin activation and cross‐bridge formation of actin and myosin in excitation–contraction coupling to elicit muscle contraction. The ability to sequencing the entirety of *RYR1* gene has dramatically increased the number of identified mutations and the awareness of many more clinical phenotypes and different modes of inheritance. Dominantly inherited *RYR1* gene mutations yielding MHS and central core pathology are usually linked with heterozygous missense mutations in hotspot regions (Klein et al. 2012; Snoeck et al. 2015). Recessive *RYR1* myopathies are usually due to a heterozygous missense variant in trans with a null or frameshift variant and can be located throughout the entirety of the coding region (Jungbluth et al. 2002; Monnier et al. 2008; Klein et al. 2012). Although a severe neonatal myopathy has been described in association with dominant *RYR1* mutations (Romero et al. 2003), patients with recessive disease often demonstrated a more severe clinical phenotype with axial and often facial weakness (Klein et al. 2012) and some with opthalmoplegia and distal weakness (Ferreiro et al. 2002; Jungbluth et al. 2005; Wilmshurst et al. 2010).

The proband's peculiar clinical features are the distal onset of upper limb weakness suggestive of a distal myopathy phenotype (Udd 2014) and the late development of jaw contracture. As in distal myopathies, our patient's weakness continues to be more severe distally than proximally. The jaw contracture, which is usually observed in early‐onset congenital myopathies, in our patient developed in his sixth decade of life. The childhood‐onset cold‐induced hand stiffness with no clinical or electrophysiological evidence of myotonia raises the possibility that this symptom may have represented early onset hand weakness. However, cold‐induced hand stiffness has been previously reported in association with *RYR1* mutations (Voermans et al. 2016). WES did not identify any other known pathogenic or presumed pathogenic variants in any gene so far known to cause distal myopathies, including *ACTA1*, recently recognized as causative of distal myopathy (Liewluck et al. 2017). The previously reported distal weakness in *RYR1*‐recessive myopathy was described only in the setting of early onset global weakness which was not our patient's main clinical feature (Ferreiro et al. 2002; Wilmshurst et al. 2010).

The patient's pathological phenotype best fits a centronuclear myopathy, being the fibers with small core‐like structures very few. The combination of clinical and morphological features is reminiscent of autosomal dominant dynamin 2 (*DNM2*)‐myopathy, although the latter predominantly affect distal lower limb and often has radial distribution of sarcoplasmic strands on NADH (Fischer et al. 2006), a morphological feature that was extremely infrequent in our patient. Because of the jaw contracture, although of late onset, we specifically searched also for associated potentially deleterious variants in beta‐tropomyosin (*TPM2*) gene and found none.

Our patient harbors a novel heterozygous likely pathogenic *RYR1* variant (c.12315_12328delAGAAATCCAGTTCC, p.Glu4106Alafs*8), which is predicted to result in reading frame change and a stop codon. It is indeed located in the central domain of the protein, between the two EF hand motifs that bind calcium, and is predicted to be truncated just upstream the second EF hand motif (Gomez et al. 2016) and the channel domain. This deleterious mutation is expected to result in a truncated nonfunctional calcium release channel or nonsense‐mediated mRNA decay. The second heterozygous *RYR1* variant (p.Arg3550Trp), which affects a highly conserved amino acid, is predicted to be damaging by SIFT, probably damaging by Polyphen and disease causing by MutationTaster, raising the possibility that this variant may contribute to the phenotype. One could also speculate that our patient's distal weakness, which was previously reported in recessive *RYR1* myopathy, although in the setting of severe generalized weakness, might signal a recessive disease, and, therefore, a contributing pathogenic role of the *RYR1* missense variant. The Arg3550Trp variant has been reported in the in the ExAC database with a MAF of 0.0002311. Not many variants in the proximity of Arg 3550 have been functionally characterized at the present time. Further studies are needed to confirm the effect of the Arg3550Trp alteration. Of interest, a dominant missense mutation (p.Ser4112Leu) located in the same region of the proband's null mutation was previously reported in a patient with neonatal‐onset centronuclear myopathy and ophthalmoparesis (Jungbluth et al. 2007). However, more commonly *RYR1*‐centronuclear myopathy is associated with recessive disease and often accompanied by ophthalmoparesis, which our patient did not have.

The proband's son's myalgia and elevated CK suggests that RYR1 p.Glu4106Alafs*8 has a dominant effect. The discrepancy in disease severity between proband and son could simply reflect intrafamilial phenotypic variability or a potential deleterious contribution from the missense *RYR1* variant in the proband. The son's younger age could also explain the current milder phenotype, especially in light of the proband's adulthood onset of the weakness.

The hemizygous missense DMD variant (p.Leu2078Phe) is accompanied by normal muscle dystrophin expression. The Leu2078Phe variant has conflicting in silico prediction as damaging by SIFT, benign by Polyphen, and polymorphism by MutationTaster. In addition to all this, the distal myopathy phenotype points away from a dystrophinopathy. We cannot entirely exclude that the DMD variant might contribute to the calf hypertrophy, but calf hypertrophy has been observed in *RYR1* myopathy (personal unpublished observation) and in rare patients with central core disease, although they had no genetic testing (Reimers et al. 1996). Our findings suggest that *RYR1* gene analysis should be included in the list of genes causative of distal myopathies. It remain to be determined if the described phenotype could be linked to the specific *RYR1* null mutation or to the combined effect of the null and missense *RYR1* variants.

## Conflict of Interest

The authors report no conflict of interest.
